# Physicochemical and Antibacterial Properties of Novel, Premixed Calcium Silicate-Based Sealer Compared to Powder–Liquid Bioceramic Sealer

**DOI:** 10.3390/jcm9103096

**Published:** 2020-09-25

**Authors:** Naji Kharouf, Youri Arntz, Ammar Eid, Jihed Zghal, Salvatore Sauro, Youssef Haikel, Davide Mancino

**Affiliations:** 1 Faculté de Chirurgie Dentaire, Université de Strasbourg, 8 rue Sainte Elisabeth, 67000 Strasbourg, France; youri.arntz@unistra.fr (Y.A.); youssef.haikel@unistra.fr (Y.H.); davidemancino@icloud.com (D.M.); 2 Institut National de la Santé et de la Recherche Médicale, Unité Mixte de Recherche 1121, 11 rue Humann, 67085 Strasbourg, France; 3 Department of Endodontics, Faculty of Dental Medicine, Damascus University, 0100 Damascus, Syria; ammarendo89@gmail.com; 4 ICube Laboratory, UMR 7357 CNRS, Mechanics Department, University of Strasbourg, 67000 Strasbourg, France; zghal@unistra.fr; 5 Laboratoire Energetique Mecanique Electromagnetisme, University of Paris Ouest, 50 rue de Sèvres, 92410 Ville d’Avray, France; 6 Departamento de Odontología, Facultad de Ciencias de la Salud, Universidad CEU-Cardenal Herrera, C/Del Pozo (s/n), Alfara del Patriarca, 46115 Valencia, Spain; salvatore.sauro@uchceu.es; 7 Department of Therapeutic Dentistry, I.M. Sechenov First Moscow State Medical University, 119146 Moscow, Russia

**Keywords:** bioceramic sealer, filling ability, antibacterial activity, physicochemical properties, calcium-silicate-based materials

## Abstract

The aim of this study was to compare the physicochemical properties, filling ability, and antibacterial activity of a premixed calcium silicate-based sealer to those of a powder–liquid bioceramic sealer. Ceraseal (CS) and BioRoot (BR) materials were analyzed using scanning electron microscopy and energy-dispersive X-ray spectroscopy at 7 and 14 d of immersion in distilled water. The filling ability of the two sealers as well as the water contact angle, solubility, flow, roughness, crystalline microstructure, pH, and compressive strength were also evaluated. The antibacterial activity was assessed through an agar diffusion as well as through direct tests. All the results were statistically analyzed using one-way or two-way analysis of variance tests. Statistically significant lower void percentages were observed for CS at 2 and 8 mm from the working length (WL) compared to those for the BR group, whilst no significant difference was observed at 5 mm from the WL. BR sealer showed higher alkaline pH, rougher surface, lower water contact angle values, lower flowability, and higher solubility compared to CS. BR showed globular and needle-like crystalline microstructure, whilst CS had globular and flower-like crystalline microstructure up to 72 h. No statistical difference was found for the compressive strength between the two sealers. BR and CS showed no antibacterial effect against *Enterococcus faecalis* after 3 h, whilst both sealers showed antibacterial capacity after 24 and 72 h. BR demonstrated higher antibacterial activity after 24 h. In conclusion, the use of bioceramic sealers may play an important role in controlling bacterial growth. Moreover, CS may have superior filling ability and lower solubility than the BioRoot sealer due to its specific chemical composition and mixing method.

## 1. Introduction

A successful endodontic treatment depends on appropriate preparation of the cavity access, suitable shaping, proper cleaning, and tridimensional filling of the root canal space [1]. It is well known that no cleaning protocol allows the eradication of 100% of bacteria in an infected root canal system [2]. Therefore, the obturation procedures must have the aim of entombing the remaining bacteria to ensure peri-apical healing [3]. To provide a tridimensional seal, the root canal filling involves the use of gutta-percha in combination with a root canal sealer. However, the incidence of voids in root canal obturation has been reported regardless of technique; this may result in the proliferation of residual bacteria and compromise the long-term outcome of root canal treatment [4]. This is particularly true in oval or irregular-shaped root canals [5]. As there is no adhesion between gutta-percha and dentin, the sealer must fill the space between the dentinal walls of root canal and the gutta-percha cone(s) [6,7]. Several endodontic sealers are available with different main composition, such as silicone, glass ionomer, epoxy resin, zinc oxide, calcium hydroxide, and bioceramic (calcium silicate) [8,9,10]. However, it has been emphasized that an excessive sealer volume may adversely influence the seal, as the majority of sealers shrink upon setting and dissolve over time. To counteract this issue, it has generally been suggested to maximize the gutta-percha volume and minimize the sealer thickness, using thermo-plasticized gutta-percha obturation techniques, including warm vertical compaction and carrier-based obturations. These techniques require specific materials and a long learning curve, which limits their use by general practitioners. More recently, the use of hydraulic condensation has been suggested since calcium-silicate-based root canal sealers (CSBRCSs) have been introduced in the market. This technique, based on the use of gutta-percha cones and CSBRCSs, does not require intracanal gutta-percha plugging, except at the level of the canal orifice. Indeed, hydraulic condensation places strong emphasis on the improved properties of CSBRCSs, especially their absence of shrinkage upon setting. Moreover, their biological and/or bioactive properties, including their biocompatibility and antibacterial activity, are also relevant once such materials are in contact with body fluids (i.e., saliva, blood, dentinal fluid) as results of its composition [11,12,13]. This technique may prove to be easier to perform and have a fairly short learning curve [14]. Some of these bioceramic sealers are constituted by powder and liquid and they must be manually mixed strictly following the manufacturer’s instructions [15]. Indeed, the manual mixing and any alteration of the powder–liquid ratio of such sealers could influence the physicochemical properties of these bioceramic cements [15,16,17]. Conversely, premixed bioceramic sealers do not have such possible issues as they come pre-dosed and do not require any manual mixing procedure. For instance, Ceraseal (CS) (Meta Biomed Co., Cheongju, Korea) is a premixed calcium-silicate-based material sealer [13], but unfortunately, there is very little information in literature on its properties and performance in vitro and in vivo. BioRoot RCS (BR) (Septodont, Saint Maur des Fossés, France) is instead a powder–liquid bioceramic sealer that requires manual mixing procedures [18]. Such bioceramic sealers have been advocated as appropriate materials for the single-cone obturation technique on [19,20], rather than for the warm technique, this latter technique may induce alteration of the physicochemical properties of the sealer [21].

It is indeed well known that appropriate physicochemical properties are recommended for these root canal sealers such as low solubility, good sealing ability, radiopacity, biocompatibility, biological potential, and antibacterial activity [13,22,23].

Thus, this study aimed at investigating and comparing some specific physicochemical properties and the filling ability of a manual-mixing and auto-mixing bioceramic sealers containing calcium silicate. Moreover, the in vitro antibacterial activity against *Enterococcus faecalis* of such bioceramic sealers was also evaluated after 3, 24, and 72 h. The hypothesis of this study was concerned with whether there would be physicochemical and/or antibacterial differences between the two tested CSBRCSs.

## 2. Experimental Section

BioRoot RCS (BR) and Ceraseal (CS) were mixed and used as according to the manufacturer’s instructions as described in Table 1.

### 2.1. Filling Ability

Sample size estimation was calculated and two groups of 10 teeth each were finally formed in order to have 80% power and an alpha error probability of 0.05.

Twenty freshly extracted first mandibular premolars, extracted for orthodontic reasons, on patients aged between 18 and 24 years old, with fully formed apices and single canal, were obtained under a patient-informed consent. The ethics committee of the medical, odontology school and Strasbourg University Hospital approved the protocol (protocol no. 2018-89).

**Figure 1:**
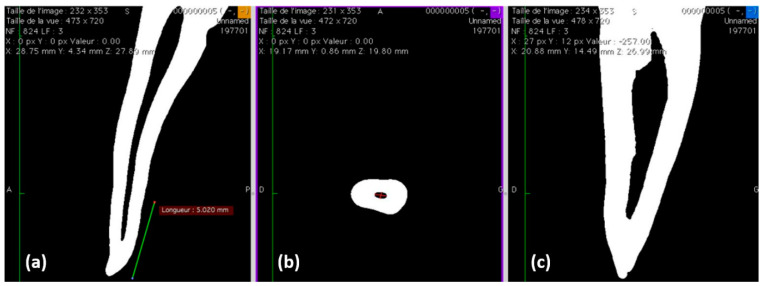
(**a**–**c**) Cone-beam computed tomography (CBCT) analysis for teeth selection to ensure the following criteria: single canal, long/short diameter (ratio > 2), and root curvature (≤20°).

After debridement of the root surface, specimens were immersed in a 1% NaOCl solution at 4 °C for 24 h and then stored in saline solution [24]. Teeth were first scanned with cone-beam computed tomography (CBCT) (Figure 1) and selected according to the following morphological parameters:

1. Single canal,
2. Long/short diameter (ratio > 2) at 5–8 mm from the apex [25],
3. Root curvature (≤20° according to the Schneider method) [26].

Cusps were flattened using silicon carbide grit papers (320 grit) mounted on a rotating polishing machine (Escil, Chassieu, France) to obtain a stable reference point and to standardize root canal length at 21 ± 1 mm.

A single and well-trained operator performed all the endodontic procedures in order to standardize the operative procedures as much as possible. A conventional endodontic cavity was prepared under an operating microscope (Zumax Medical Co., Ltd., Jiangsu, China). Canal scouting was performed in all specimens with a #10 K-file. The working length (WL) was established using a #10 K-file under high magnification by subtracting 0.5 mm from the length at which the tip of the instrument was visible at the apical foramen. Subsequently, the apices were sealed with a cyanoacrylate glue in order to achieve a closed system [24].

The teeth were then included in polyvinylsiloxane (PVS) impression materials up to the cemento-enamel junction (CEJ). A mechanical glide path enlargement was performed using ProGlider (Dentsply Sirona Endodontics, Ballaigues, Switzerland) powered by an endodontic motor (X-smart-IQ motor, Dentsply Sirona, Ballaigues, Switzerland) in a continuous clockwise rotation at 300 rpm and 2.5 Ncm. Root canal shaping was performed sequentially using ProTaper Next X1 (tip size = 0.17 mm, taper = 0.04) and X2 (tip size = 0.25 mm, taper = 0.06) with the same parameters (Dentsply Sirona, Ballaigues, Switzerland).

Each canal was irrigated using the same final irrigation protocol based on 2.5 mL of physiological saline over 90 ± 5 s, 5 mL of 17% EDTA solution over 120 ± 10 s, 2.5 mL of physiological saline over 90 ± 5 s, 5 mL of a 6% NaOCl over 120 ± 10 s followed by a final rinse with 2.5 mL of physiological saline over 90 ± 5 s. Final irrigation solutions were activated using an EndoUltra cordless device (MicroMega, Besancon, France) with the Ni–Ti tip (15/0.02) oscillating at 40 KHz.

The specimens were randomly divided in two equal groups (*n* = 10) for obturation with two single-cone techniques using a standardized amount of sealer (0.230 ± 0.010 g) and a Protaper Next X2 Conform Fit gutta-percha cone:

- Single-cone technique (SC1) with Ceraseal (Meta Biomed Co., Cheongju, Korea)
- Single-cone technique (SC2) with BioRoot (Septodont, Saint Maur des Fossés, France)

If necessary, the gutta-percha point was trimmed back until tug-back and was achieved at a WL of 0.5 mm.

With regard to the SC1 technique, the cement was delivered into the canal according to the manufacturer′s instructions. Whereas, for the SC2 technique, the sealer was delivered into the root canal with a lentulo spiral (Dentsply Sirona, Ballaigues, Switzerland) to the working length.

The specimens were stored in water at 37 °C for 72 h. After the storage period, three sections were performed perpendicularly to the longitudinal axis of the tooth root at 2, 5, and 8 mm from the apex with a diamond saw (Walter EBNER, Le Locle, Switzerland). Subsequently, 1200, 2400, and 4000 SiC abrasive papers were used, under continuous water irrigation, to polish the surface of the specimens. They were first observed using an optical numeric microscope (KEYENCE, Osaka, Japon) and then analyzed using the VHX-5000 communication software (KEYENCE, Osaka, Japon) to measure the total area of the voids at 2, 5, and 8 mm from the apex. The void percentages were calculated by dividing the area of the voids measured during the analysis with the total area of the root canal of each specimen. Finally, the polished surfaces were treated with 37% phosphoric acid (5 s) and 2.5% NaOCl (3 min) and thoroughly rinsed with distilled water for 30 s in order to eliminate the smear layer resulting from the polishing process and to visualize the sealer infiltrations in dentinal tubules (tags) [27]. The specimens were dehydrated in a graded series of ethanol solutions and sputter-coated with a gold–palladium alloy (20/80 weight %) using a Hummer JR sputtering device (Technics, CA, USA). These were analyzed using a Quanta 250 FEG (field emission gun) scanning electron microscope “SEM” (FEI Company, Eindhoven, The Netherlands) with an electron acceleration voltage of 10 kV to verify the presence of sealer infiltrations (tags) into dentinal tubules.

### 2.2. pH Measurements of the Water in Contact with the Sealers

Five polyethylene tubes measuring 1.6 mm in diameter and 10 mm in height [28] were filled with freshly prepared sealers using a spatula and an endodontic K-file (Micro-Mega, Besançon, France). Residues of the sealer were cleaned from the external walls, and each specimen was immersed in a glass vial with 10 mL of distilled water (initial pH = 6.8) (water system Elix^®^ Millipore, Merck Chimie SAS, Ile-de-France, France). The pH measurements were performed at 24 ± 2 °C after incubation at 37 °C for 3, 24, 72, and 168 h. Before each pH measurement time, the pH meter was calibrated with standard solutions at pH 4, 7, and 10 (Hanna Instruments, Lingolsheim, France). The pH meter electrode was rinsed with distilled water to eliminate contamination from the previous solution.

### 2.3. Solubility and Flow Tests

Fifteen specimens for each tested material were prepared as reported by Schäfer [23] and analyzed by using the procedure described in ISO 6876:2001. Stainless steel ring molds (internal diameter of 20 ± 0.1 mm and a height of 1.6 ± 0.1 mm) were first cleaned with acetone in an ultrasound bath for 15 min and then weighed three times (accuracy ±0.0001 g; Ohaus, Pioneer^®^ Analytical, Nänikon, Switzerland) before sealer injection, and the mean value was recorded. They were placed on a glass plate, and the sealers were plugged into the molds avoiding air entrapment. All the specimens were stored in a dark container at 37 °C and 95% relative humidity for 72 h. The filled molds were weighed again 3 times (accuracy ±0.0001 g) before aging immersion periods. After the two immersion periods (1, 7, and 14 days; *n* = 5 from each sealer for each time point) in 50 mL of distilled water at 37 °C, the specimens were removed from distilled water, washed gently with 5 mL of distilled water, and dried at 37 °C for 24 h. Thereafter, the weight of each specimen was assessed three times, and the mean was recorded as the final weight. The difference of mass between the initial weight (before immersion) and the final weight was recorded as a percentage to determine the solubility percentage of each sealer.

Flow test was also performed by using the procedure described in ISO 6876/2001: 50 µL of each mixed sealer was dispensed on a separate glass plate (40 × 40 × 5 mm). Then a second glass plate was carefully placed on top of the sealer, along with a weight of 100 g applied centrally on top of the second glass plate. After 10 min of mixing, the maximum and minimum diameter of the compressed sealer were measured using a digital caliper (Dexter, Elkhart, Indiana, United States). The mean diameter was calculated, and the test was repeated if the difference between the two measurements reached more than 1 mm.

### 2.4. Scanning Electron Microscope (SEM) and Energy-Dispersive X-ray (EDX) Analysis

Twenty specimens for each sealer tested in this study were plugged into molds, as previously described. They were immediately stored in a dark container at 37 °C with 95% humidity for 24 and 72 h; then they were kept in distilled water at 37 °C for 7 and 14 d. Subsequently, five specimens of each sealer, for each time point, at 1, 3, 7, and 14 d, were prepared and submitted to SEM observations and EDX analysis. The sealers were removed from their molds and mounted onto aluminum SEM stubs. These were sputter-coated with gold–palladium (20/80) and their morphological and chemical characteristics were analyzed using a SEM at a magnification of ×4000. EDX analysis was performed with a working distance of 10 mm and an acquisition time of 60 s. The weight percentages of chemical elements of the surfaces of the specimens of the different sealers were attained.

### 2.5. Surface Roughness and Water Sorption Tests

Five specimens for each group were prepared using Teflon molds (diameter: 16 mm; height: 3 mm). They were glued on a glass slide and stored in hermetic boxes at 37 °C in 100% relative humidity for 48 h.

The Atomic Force microscopy (AFM) was performed using the Bioscope Catalyst (Bruker Inc., Santa Barbara, CA, USA) equipped with MLCT (Metal-to-Ligand Charge-Transfer) cantilevers (0.7 N/m) (Bruker Inc., Santa Barbara, California, United States). Images were captured at a specific resolution (256 × 256) with a scan rate of 1 Hz (field of view of 10 × 10 µm). The roughness (Ra) was calculated using a shareware software GWYDION on five different representative regions of interest.

After measuring the surface roughness, a device to measure contact angle (Attension Theta, Biolin Scientific, Götenborg, Sweden) was used to evaluate the absorption time of a 5 µL drop of distilled water into the cement surface. In turn, a drop of distilled water was placed using a needle with an inner diameter of 0.7 mm onto the surface of each specimen. The contact angle was recorded after 10 s of deposition, and the mean left and right contact angles were calculated to obtain the final contact angle. A video was recorded for each test using a horizontal camera to track the profile of the water drop.

### 2.6. Compression Tests

Ten specimens were created for each tested material using a Teflon mold (inner diameter: 3 mm; in height: 3.58 mm) [29]. They were immersed for 24 h in distilled water at 37 °C, removed from their molds, and checked using a Zumax microscope (Zumax medical, Suzhou New District, China) at ×10 magnification in order to detect the presence of voids or any other defect; such specimens were discarded before any further process.

All specimens were tested through a uniaxial compression test to determine the compressive strength of the cement and the maximum load. The test was performed using the universal tensile Instron 3345 (Instron^®^, Norwood, MA, USA) device instrumented with a 1 kN cell force (Class 0.5 following ISO 7500-1) and with a displacement sensor. A constant crosshead speed of 0.5 mm/min was used. The compressive strength was calculated in megapascals (MPa) according to the formula:

σ_c_ = 4P/πD^2^ (1)

where P is the recorded load during the test and D is the initial sample diameter.

### 2.7. Antibacterial Activity

*Enterococcus faecalis* (*E. faecalis*, ATCC 29212) was cultured according to its manufacturer’s instructions using Brain Heart Infusion “BHI” (Panreac Applichem ITW Reagents, Hessen, Germany). The turbidity of BHI containing *E. faecalis* was adjusted to optical density (OD) at a wavelength of 600 nm, OD_600(nm)_ = 0.5. The initial number of cells/mL was 2.5 × 10^8^ CFU/mL.

#### 2.7.1. Agar Test

The agar diffusion test was performed similarly to ref. [30] using six identical agar-filled Petri dishes containing 25 mL of BHI. Three plates were used to evaluate the antibacterial activity of the different sealers. One hundred microliters of bacterial medium was spread homogeneously onto the Petri dishes. Five wells in each Petri dish, 3 mm in diameter and 3 mm in depth, were made with an adapted punch by removing the agar. Two wells were each filled with the different sealers and the fifth was filled with 21 μL of 20% chlorhexidine (CHX) (ref.18472510, Sigma Aldrich, Saint-Quentin-Fallavier, France) to serve as a negative control. Two further plates were left unfilled and served as positive control, while one plate was filled with the sealers, but without using any bacterial medium (negative control) in order to verify the cleanness of the sealers used.

All the agar Petri dishes were incubated at 37 °C for 48 h under aerobic conditions. The same operator measured the inhibition zones after 48 h of incubating. The inhibition zone was determined as half the inhibition zone diameter minus the well diameter. The inhibition zone was measured in millimeters using a digital caliper (Dexter, Elkhart, IN, USA) and was recorded for each sealer.

#### 2.7.2. Direct Test

Three specimens for each sealer were prepared as previously described in the section for the compression strength evaluation. Each sample was placed in a 2 mL Eppendorf tube (Trefflab, Degersheim, Switzerland). One milliliter of bacterial medium was injected in each Eppendorf tube. In the positive control, the bacterial medium was inserted into the Eppendorf tube without any sealer. Subsequently, all the Eppendorf tubes of this test were incubated for 3, 24, and then 72 h at 37 °C under constant stirring. After each incubation period, 10 μL from each Eppendorf tube was assessed by 10-fold serial dilution up to 10^5^ in BHI, and then 100 μL of the dilution was spread homogeneously onto the plate. The plates were incubated at 37 °C for 24 h. After incubation, colonies on the plate were counted and their CFU/mL mean was determined.

### 2.8. Statistical Analysis

The collected data were analyzed with SigmaPlot release 11.2 (Systat Software, Inc., San Jose, CA, USA). Shapiro–Wilk test was used to verify the normality of data. However, when the normality was not verified thus, Kruskal–Wallis test (one-way analysis of variance on ranks) was used. The proportions of void areas corresponding to each filling technique, the values for roughness, compressive strength analysis, antibacterial activity, and contact angle measurements were compared by one-way ANOVA. A two-way ANOVA test was applied to determine whether significant differences existed in the pH values of the different solutions in contact with the two sealers and the solubility measurements at 7 and 14 days. A statistical significance level was set at α = 0.05.

## 3. Results

### 3.1. Filling Ability at 2, 5, and 8 mm from the Apex

**Figure 2:**
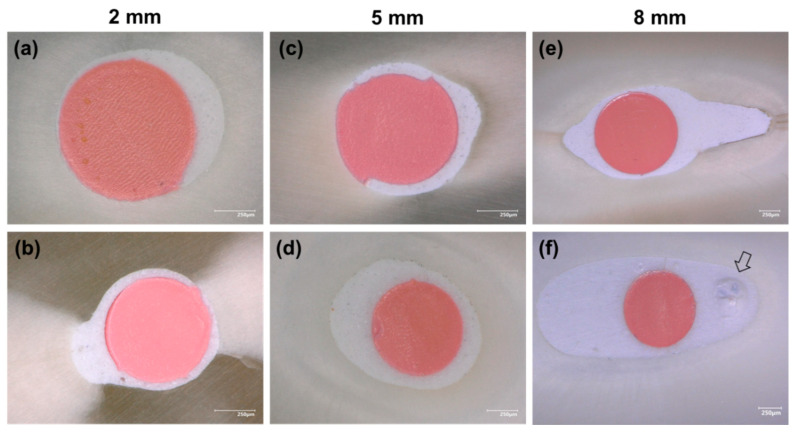
Digital microscope images. (**a**) Ceraseal, (**b**) BioRoot at 2 mm from the apex (200× magnification); (**c**) Ceraseal, (**d**) BioRoot at 5 mm from the apex (200× magnification); (**e**) Ceraseal, (**f**) BioRoot at 8 mm from the apex (100× magnification), showing an internal void (closed pores) (arrow).

Gutta-percha-filled areas, sealer-filled areas, and void areas were observed and measured at 2, 5, and 8 mm from the working length (Figure 2). All measurements were expressed as the mean ± standard error percentages (*n* = 10) (Table 2). Statistically significant lower void percentages were observed for Ceraseal at 2 and 8 mm from the working length compared to those for the BioRoot group (*p* < 0.05), whilst no significant difference was observed at 5 mm from the working length between BR and CS (*p* > 0.05).

**Figure 3:**
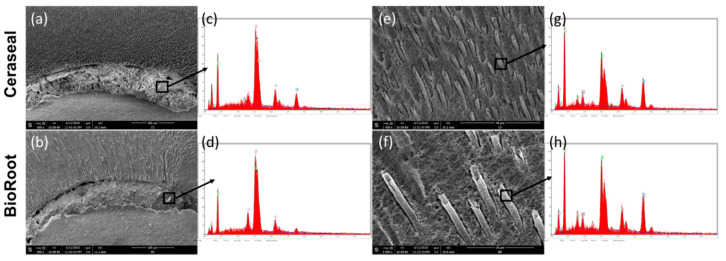
Representative photos of scanning electron microscopy of sectioned root surfaces filled with (**a**,**c**,**e**,**g**) Ceraseal and (**b**,**d**,**f**,**h**) BioRoot. (**a**,**b**) Sealer–dentin interface; (**e**,**f**) infiltrations into dentinal tubules; (**c**,**d**) chemical analysis of the sealer in root canal; (**g**,**h**) chemical analysis of the infiltrations into dentinal tubules.

Figure 3a,b shows the sealer–dentin interfaces. Regarding sealer infiltration into the dentinal tubules, tags were observed at WL-5 and -8. The dentinal tubules were occluded by hollow tags in those areas (Figure 3e,f). The chemical composition of the tags and the filled sealer are shown in (Figure 3c,d,g,h).

### 3.2. pH Measurements of the Water in Contact with the Sealers

**Figure 4:**
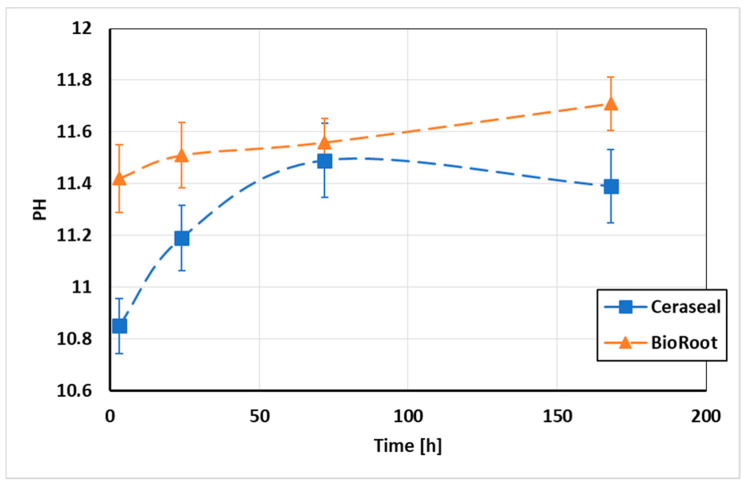
pH changes of water in contact with the sealer materials as a function of time for one week.

The mean pH values of distilled water in contact with the different sealer materials are shown in Figure 4. The two sealer specimens showed an alkaline pH. Significant higher pH values were observed for the BioRoot specimens after 3, 24, and 168 h of immersion in distilled water (*p* < 0.05), whilst at 72 h, no significantly difference was found between Ceraseal and BioRoot (*p* > 0.05).

### 3.3. Solubility and Flow Tests

The mean and standard deviation of solubility (wt.%) values of the tested materials are presented in Table 3. BR and CS sealers were soluble during the evaluation period. Significantly higher solubility percentages were reported for BR samples than for CS samples at the different time points (*p* < 0.05), except for 1 d (*p* > 0.05). The BR sealer exhibited lower flow properties than the CS sealer (*p* < 0.05) (Table 3).

### 3.4. Scanning Electron Microscope (SEM) and Energy-Dispersive X-ray (EDX) Analysis

**Figure 5:**
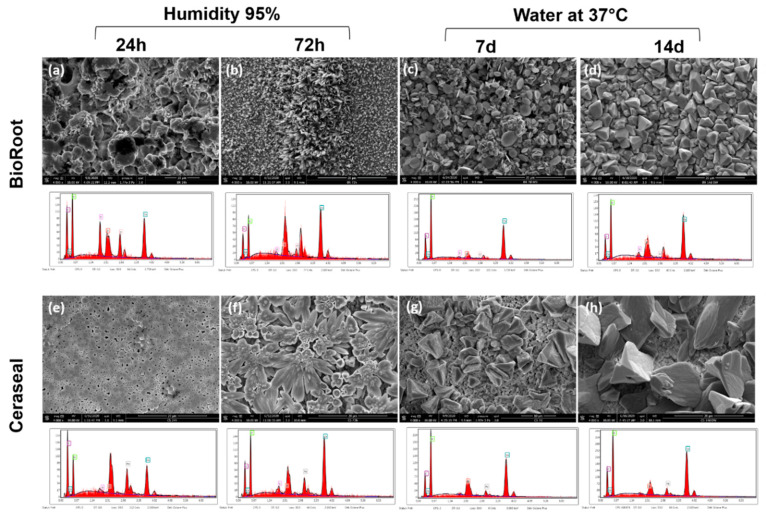
Representative scanning electron microscope images at 4000× magnification (**a**–**h**) and energy-dispersive X-ray (EDX) spectrums. The morphology and EDX spectrum observed for the BioRoot (**a**–**d**) and Ceraseal (**e**–**h**) surfaces after 24 and 72 h in humidity, 7 and 14 d in water at 37 °C.

The crystalline structures of BR and CS sealers with their EDX spectra are shown in Figure 5. During the initial setting time, BioRoot surface mostly showed a globular and needle-like appearance, at 24 and 72 h (Figure 5a,b). Ceraseal sealer showed a combination of globular and flower-like crystalline structures, at 24 and 72 h (Figure 5e,f). After 7 and 14 d of immersion in water at 37 °C, BR and CS showed cubical crystallites (Figure 5c,d,g,h). BR cubical crystallites were smaller and more numerous than CS cubical crystallites after 7 and 14 d. The mass percentages of the main chemical elements of BR and CS surfaces are shown in Table 4. The mass percentage of Ca was higher for BR rather than for CS, for all the testing periods.

### 3.5. Surface Roughness and Water Sorption Tests

**Figure 6:**
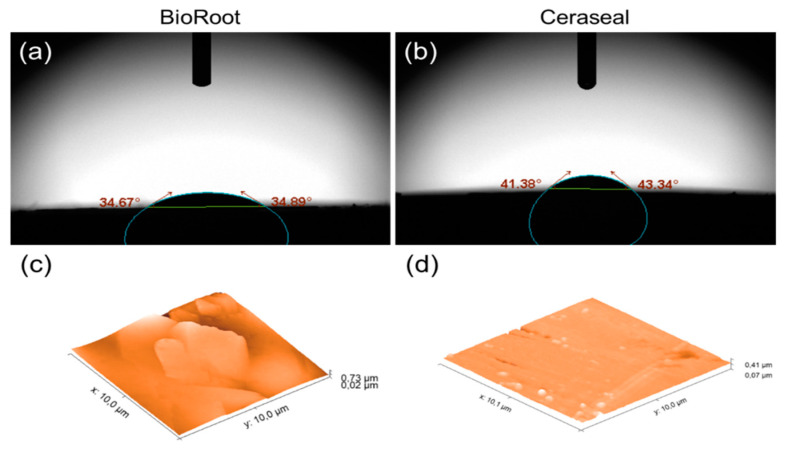
Visual aspect of a water droplet (5 µL in initial volume) deposited onto different sealer surfaces (**a**) BioRoot; (**b**) Ceraseal, 10 s after its deposition. AFM micrographs (3D view, 10 µm^2^) of the BioRoot surface (**c**) and Ceraseal surface (**d**).

The mean values and standard deviation of contact angle and absorption time for water (5 µL) on the surface of tested sealers are shown in Table 5. Significant lower values were observed for BioRoot specimens after 10 s of drop deposition (*p* < 0.05). A better spreading of distilled water was observed for all BioRoot specimens (Figure 6a), which also appeared rougher (Figure 6c) than the specimens created with Ceraseal (*p* < 0.05) (Figure 6b,d and Table 5).

### 3.6. Compression Tests

There was no significant difference in compressive strength between BioRoot (48.35 ± 22.10 MPa) and Ceraseal (55.59 ± 4.05 MPa) after 24 h in water (*p* > 0.05). However, an important increase of dispersion of compressive strength values was observed in the BioRoot group.

### 3.7. Antibacterial Activity

#### 3.7.1. Agar Diffusion Test (ADT)

**Figure 7:**
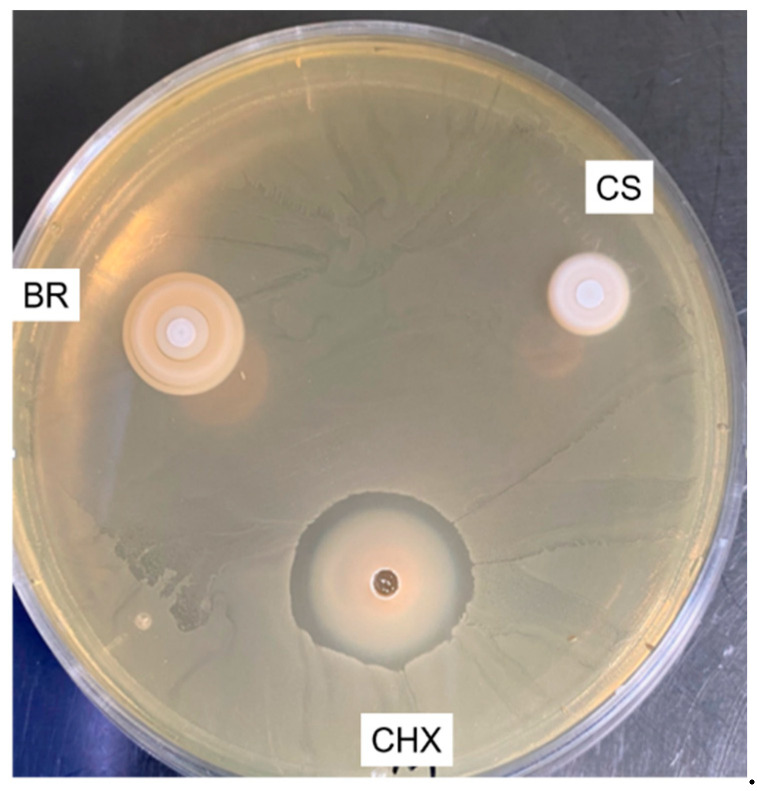
The inhibition zones around the different sealers and chlorhexidine (CHX) using agar diffusion test. BioRoot (BR) and Ceraseal (CS) materials infiltrated into agar materials.

Chlorhexidine induced the formation of a high inhibition zone (8 ± 1 mm), whilst both bioceramic sealers were not able to create any clear inhibition zone (Figure 7).

#### 3.7.2. Direct Test

**Figure 8:**
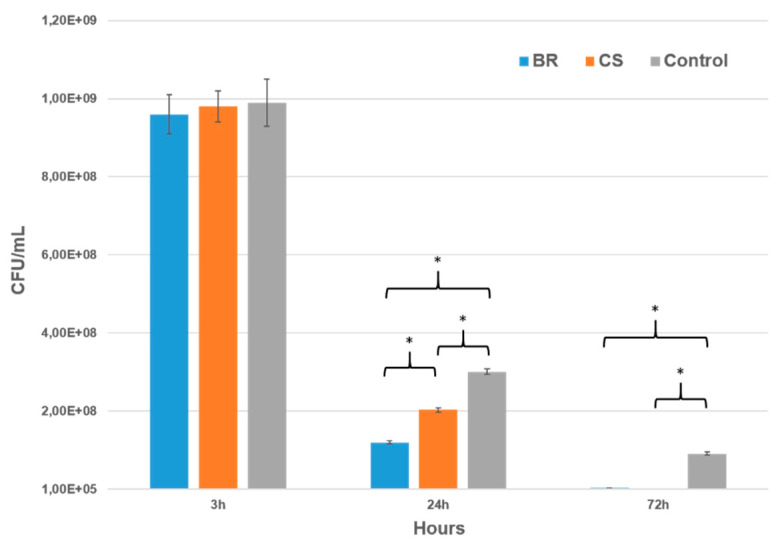
Number of CFU (Colony Forming Unit) of *Enterococcus faecalis* in the presence of BioRoot, Ceraseal, and control group after 3, 24, and 72 h of culture; (* *p* < 0.05).

This test was used to evaluate the effect of each sealer on the number of surviving bacteria after 3, 24, and 72 h of direct contact. Both sealers showed no antibacterial activity after 3 h of contact. However, BR sealer had a higher bactericidal effect (60%) after 24 h compare to CS, which killed only 32% of bacteria (Figure 8) (*p* < 0.05). After 72 h, both BR and CS killed more than 95% of *E. faecalis*.

## 4. Discussion

The main advantage of a premixed calcium-silicate root canal sealer consists in having a homogeneous mixture with no concern related to the alteration of powder/liquid ratio [15,31]. Indeed, any alteration of the powder/liquid ratio during the mixing procedure may cause alteration of the compressive strength [32], the solubility [33], and the bond strength of endodontic cement to dentin [34]. One more important advantage is its user-friendly and non-time-consuming nature [15]. A successful endodontic treatment depends on several aspects such as, shaping of the root canal system, cleaning, and a 3D root canal obturation able to provide an optimal coronal seal, reduce apical leakage, and entomb the remaining bacteria [24,35]. As far as we know, there is no information on the filing ability, physical properties, and antibacterial activity of the Ceraseal root canal sealer. Instead, biocompatibility, bioactivity, and ion release of CS sealer were already studied by López-García et al. [13].

In this study, the filing ability of BioRoot and Ceraseal sealers using the single-cone technique was evaluated at 2, 5, and 8 mm from the WL using an optical digital microscope, in order to evaluate the void percentages and interfacial gaps in apical, middle, and coronal parts of the root canal. Significantly lower void percentages were observed for the Ceraseal sealer at WL-8 mm and WL-2 mm (*p* < 0.05), whilst no significant difference was found at WL-5 mm between the two sealers. Accordingly, Viapiana et al. [36] highlighted the important amount of void percentage of BioRoot. The single-cone technique should be performed using calcium-silicate-based sealers in order to reduce the formation of voids, especially in the coronal third of the root canal [20]; this would also avoid the changes in sealer properties when it is used with a warm obturation technique [21]. The main void percentages observed for BioRoot at WL-8 mm were inside the filling materials “closed pores” (Figure 2f). In general, the voids observed in the specimens created with BioRoot at 8 mm were the result of internal porosities within the filling material; these are defined as “closed pores” (Figure 2f, arrow). No closed pores were observed for CS samples. At WL-2 mm, BioRoot had more interfacial void percentages or “open pores” than the Ceraseal sealer (Table 2). However, it was reported that interfacial voids (open pores or gaps) allowed leakage, whilst internal voids (inside filling material) have no impact on the overall obturation [37]. Indeed, interfacial gaps have been advocated to serve as a pathway for microorganisms for the reinfection the root canal system from the coronal, as well as from the apical side [38,39]. This observation may also be related to the results of the flow test attained in our study, where CS presented significant higher flowability (Table 3), as well as a higher filling ability than the BR sealer. Viapiana et al. [36] reported that the filling ability of a sealer could be related to its physical properties such as setting time and flow properties.

The penetration of the sealers into the dentinal tubule may represent an important factor to entomb the bacteria [24] and to favor an interaction between the sealer materials and the dentinal fluid; this may induce the formation of bioactive mineral crystals inside the dentinal tubules [40,41]. The sealers, BR and CS, showed intra-tubular infiltrations (tags) at 5 and 8 mm. Such tags presented a characteristic hollow shape, but with no presence of silica or zirconia in their chemical composition (Figure 3g,h). Conversely, the sealer materials in the root canal contain silica and zirconia (Figure 3c,d). This observation may be explained considering two hypothesis. Firstly, silica and zirconia elements were not able to fill the tubules and stayed in the sealer structure inside the root canal, or as a second option, these elements were present in very small amount so that the EDX device could not detect their presence. The same hollow tags were observed in the study of Kim et al. [42] when they evaluated the mineral trioxide aggregate (MTA)–dentin interfaces. The maximum tag penetration was 626 µm observed for CS sealer at WL-8 mm, whilst it was 480 µm for the BR sealer. McMichael et al. [43] studied the dentinal tubule penetration of tricalcium silicate using a confocal laser microscope. They reported that the tags of tricalcium silicate at 5 mm from the apex using single-cone technique have a depth of 2 mm into the dentinal tubules. The penetration depth of sealer materials into the dentinal tubules depends on the flowability and working time of the sealer [36], as well as on dentin treatment/irrigation and patency of the entire root canal system [44,45].

The pH change of distilled water in contact with BR and CS sealers was evaluated. Alkaline pH plays a key role as it may enhance the antibacterial activity of a material [37], improve the healing process, neutralize the lactic acid from the osteoclast [46,47], and increases the deposition of mineralized components [48]. The current study showed that both BR and CS increased the pH of the storage media (Figure 4). Higher pH values were observed for BR rather than for CS. The prolonged alkalinity of the bioceramic sealer matched the increase in solubility [47], which was more important for BR than for the CS group. The pH values were evaluated at different time points in order to analyze and compare the alkalization activity of each sealer over time. The pH measurement times were chosen in order to link their results with the antibacterial effect of both sealers. Both BR and CS presented a high solubility after 1, 7, and 14 d in distilled water (Table 3). The solubility of root canal sealers should not exceed 3% mass after 24 h in water according to ISO specification 6876:2012. The solubility of both sealers was more than 3% after 24 h. Our results are in accordance with previous studies that reported such a high solubility of BR after 1 [47], 7, and 14 d [49]. High solubility may be responsible for creating gaps between the sealer materials and the root canal dentin, causing loss of the sealing ability and a pathway for microorganism [50]. However, the higher solubility of CSBRCSs indicates high levels of Ca^2+^, which is an expression of bioactivity. Further future studies should determine the equilibrium point between solubility and bioactivity.

The microstructural crystalline formation during the initial setting time in 95% humidity and after 7 and 14 d in water was observed using an SEM. The SEM and EDX time points (7 and 14 d) were chosen to visualize the sealer surfaces according to the time points which were chosen for the solubility test (7 and 14 d). Each sealer had a different crystalline type (Figure 5). However, CSBRCSs are able to produce calcium hydroxide once it is in contact with water [11]. This calcium hydroxide is mainly responsible for the alkalinization of the storage media [11]. Both sealers showed crystalline disposition on the surface, which had high calcium peaks (Figure 5) in the EDX analysis. Camilleri [11] studied the hydration kinetics of tricalcium silicate cement, reporting that the hydration of tricalcium silicate with water forms calcium silicate hydrate and calcium hydroxide. We believe that the smaller and more numerous crystals formed on BR surface are more soluble in water than the larger CS crystals, owing to a greater surface-to-volume ratio [51]. However, our hypothesis is that all these factors could play an important role in the solubility and pH average.

The sorption test of BR and CS was evaluated using a contact angle measurement, which is an indicator of the wetting behavior of a solid material (sealer) and a liquid (water). The profile of 5 µL drops of distilled water on each sealer surface was evaluated. BR sealer had lower contact angle values and less sorption time than CS group. Lower values of contact angle indicate that the substrate is well wet and has more surface free energy than higher that with a contact angle [52]. Therefore, it appears that the surface free energy of BioRoot is relatively higher than that of the CS surface. This could be explained by the fact that the BR surface was rougher than the CS surface. The surface roughness is one of the main factors that may affect the contact angle measurement [52]. Moreover, a rougher surface can also provide a superior biocompatibility [53]. Indeed, it seems that BR sealer increases the adhesion, the cellular attachment, and protein adsorption [54]. The water contact angle depends on the chemical surface composition and on the materials roughness. Whatever the considered wetting model (Wenzel or Cassie–Baxter) [51]. Our results (Table 5) showing that BR has a contact angle of (34.76 ± 1.26) degrees, which is smaller than the contact angle of CS (42.38 ± 0.91) degrees and simultaneous BR has a higher roughness, are in agreement with the Wenzel model, which gives an apparent cosine of the contact angle as a function of the roughness and the cosine of real contact angle. The Wenzel model implies that rough and hydrophilic surfaces appear more hydrophilic than the corresponding flat surfaces [51].

The compressive strength of an endodontic sealer was appraised as unimportant and having no essential properties; as such, materials are not subjected to high compressive stress in the root canal [55]. Conversely, the compressive strength may play an important role in reinforcing the instrumented root canal, the tooth, as well as the resistance of displacement of the cone during and after cone placement [56]. However, CS and BR showed no significant difference regarding their compressive strength values (*p* > 0.05), although BR had a high dispersion in its values probably due to the presence of important internal voids.

CSBRCSs, which are considered bioceramic materials [12], were introduced to the market thanks to their bioactive and biological properties [57] such as antibacterial activity [58], osteogenesis potential, and biocompatibility [59]. Thus, the present study evaluated the in vitro antibacterial activity of BR and CS against *E. faecalis*. *Enterococcus faecalis* is a Gram-positive facultative anaerobe and is able to grow in the presence or absence of oxygen [60,61]. *E. faecalis* was selected in our research because it is a microorganism and predominant bacteria found in persistent endodontic infections and in failed root canal treatment cases [58,61]. The most common tests for evaluating antimicrobial effect are the agar diffusion and the direct contact tests [29,62]. The agar test was not successful in evaluating the in vitro antibacterial activity due to the fact that the sealer materials infiltrated the agar substrate, thus, hiding the inhibition zones (Figure 7). Huang and collaborators [62] reported that the solubility and diffusibility of dental sealers into agar materials influence the agar test results. The diffusion of the BR sealer into the agar was notably larger than that of CS, it could be due to the fact that BR is more soluble (Table 3), thus, it could infiltrate in the agar more than CS. In the direct test, the BR sealer had a higher antibacterial capacity than CS after 24 h; this may be due to its higher pH, which plays an important role in the antibacterial activity [22]. After 72 h, the two sealers showed a high antibacterial activity (killed bacteria more than 95%) compared to the control group, maybe due to their strong pH and the high degree of Ca^2+^ release [13,18]. In addition, a part of the bacteria may die due to the depletion of the nutrients, which was seen in the control group between 3 and 72 h.

The images of the optical numeric microscope (low resolution) and scanning electron microscopy (high resolution) of polished sections have limitations, i.e., only a small number of sections could be examined using these destructive techniques. Thus, data derived from optical microscope and SEM examination of interfacial gaps and voids are only representative of sectioned canal levels examined. The evaluation of antibacterial activity using ADT had a limitation due to the fact that the sealer materials were infiltrated in the agar, thus, hid the inhibition zones.

Interfacial gaps and voids in filled root canals could be clinically relevant; they could theoretically act as bacterial survival shelter and their by-products may have a negative impact on the clinical outcome. Similarly, sealer antibacterial activity is clinically relevant because it is well know that cleaning/disinfection procedures do not eradicate 100% of bacteria in an infected root canal system.

Further studies are required to evaluate the mineralogical characteristics of CS when it is in contact with different solutions using X-ray diffraction, the bond strength of CS to the dentin surface, and the setting time of CS sealer. However, this is the first study to evaluate the capacity of the Ceraseal to fill the root canal, its antibacterial efficacy against *E. faecalis*, some physical properties, and its mineralogical characteristics in distilled water.

## 5. Conclusions

Within the limitations of this study, we can conclude that the use of bioceramic sealers may play an important role in controlling bacterial growth. Moreover, CS may have superior filling ability and lower solubility than BioRoot sealer due to its specific chemical composition and mixing method. However, further clinical trials and in vivo studies are necessary to confirm these latter outcomes.

## Figures and Tables

**Table 1:** Manufacturer, composition, and manipulation of the tested materials [13,18].

Materials	Manufacturer	Composition	Mixing
**Ceraseal (CS)**	Meta Biomed Co., Cheongju, Korea	Calcium silicates, zirconium oxide, thickening agent	Premixed (auto-mixing tip)
**BioRoot RCS (BR)**	Septodont, Saint Maur des Fossés, France	Powder: tricalcium silicate, zirconium oxide, and excipients. Aqueous solution: calcium chloride and excipients	Powder (1 scoop) and liquid (5 drops)

**Table 2:** Gutta-percha filling area (GPFA), sealer area, and void (interfacial and intracanal voids) percentages in the samples at 2, 5, and 8 mm from the working length.

Levels	Groups	GPFA (%)	Sealer Area (%)	Voids (%)	
				Interfacial	Intracanal
**2 mm**	**CS**	71.88 ± 4.42	28.01 ± 4.37	0.11 ± 0.19	0
	**BR**	67.86 ± 4.30	31.29 ± 4.36	0.85 ± 0.84	0
**5 mm**	**CS**	60.72 ± 6.71	39.12 ± 6.84	0.16 ± 0.36	0
	**BR**	60.47 ± 6.52	39.28 ± 6.36	0.13 ± 0.25	0.12 ± 0.32
**8 mm**	**CS**	51.28 ± 2.02	48.72 ± 2.02	0	0
	**BR**	51.70 ± 3.97	46.87 ± 4.12	0.31 ± 0.69	1.12 ± 1.54

CS, Ceraseal; BR, BioRoot.

**Table 3:** Solubility (*n* = 5) of Ceraseal and BioRoot in distilled water at 37 °C and flow test (*n* = 5). Data expressed as mean percentages with standard deviation.

Solubility			
Group\Time	1 Day	7 Days	14 Days
**Ceraseal (%)**	10.72 ± 2.03	12.61 ± 0.44	13.92 ± 0.66
**BioRoot (%)**	12.71 ± 1.76	14.61 ± 0.64	16.51 ± 0.67
**Flow**			
**Ceraseal (mm)**	27 ± 0.4		
**BioRoot (mm)**	18 ± 0.3		

**Table 4:** The mass percentages of the main element compounds of BioRoot and Ceraseal surfaces in 95% humidity after 24 and 72 h, 7 and 14 d in water at 37 °C.

Sealer	Element (%)	24 h	72 h	7 d (Water)	14 d (Water)
**BR**	**Ca**	35.69 ± 6.27	40.94 ± 9.76	43.35 ± 9.73	51.36 ± 4.57
	**Zr**	6.25 ± 2.24	8.81 ± 1.78	1.71 ± 0.30	4.32 ± 1.93
	**Si**	1.89 ± 2.04	1.95 ± 1.20	0.77 ± 0.65	0.69 ± 0.42
	**Cl**	11.75 ± 2.44	3.63 ± 0.47	0.31 ± 0.09	0.58 ± 0.51
**CS**	**Ca**	24.56 ± 12.23	39.59 ± 6.17	29.56 ± 10.43	44.10 ± 4.52
	**Zr**	1.04 ± 0.68	4.66 ± 2.88	24.08 ± 13.11	5.98 ± 2.86
	**Si**	0.41 ± 0.14	0.85 ± 0.55	1.24 ± 0.91	0.60 ± 0.17

**Table 5:** Evolution of the time required for the two sealers to engulf a 5 µL water droplet, the profile of the water drop after 10 s of deposition on the sealer surface, and the surface roughness mean values and standard deviations.

	BioRoot	Ceraseal	*p* Values
**Absorption time (min)**	15 ± 1	18 ± 1.4	0.002
**Contact angle (ϴ°)**	34.76 ± 1.26	42.38 ± 0.91	≤0.001
**Surface roughness (Ra, nm)**	67.81 ± 21.32	22.83 ± 3.92	0.008
